# Patterns of diagnostic imaging and associated radiation exposure among long-term survivors of young adult cancer: a population-based cohort study

**DOI:** 10.1186/s12885-015-1578-1

**Published:** 2015-09-03

**Authors:** Corinne Daly, David R. Urbach, Thérèse A. Stukel, Paul C. Nathan, Wayne Deitel, Lawrence F. Paszat, Andrew S. Wilton, Nancy N. Baxter

**Affiliations:** 1 Department of Surgery, Li Ki Shing Knowledge Institute, St. Michael’s Hospital, Toronto, Canada; 2 Department of Surgery, University Health Network, Toronto, Canada; 3 Institute for Clinical Evaluative Sciences, Toronto, Canada; 4 Institute of Health Policy, Management and Evaluation, University of Toronto, Toronto, Canada; 5 Division of Haematology/Oncology, The Hospital for Sick Children, Toronto, Canada; 6 Department of Radiology, St. Michael’s Hospital, Toronto, Canada; 7 Department of Radiation Oncology, Sunnybrook Health Sciences Center, Toronto, Canada

## Abstract

**Background:**

Survivors of young adult malignancies are at risk of accumulated exposures to radiation from repetitive diagnostic imaging. We designed a population-based cohort study to describe patterns of diagnostic imaging and cumulative diagnostic radiation exposure among survivors of young adult cancer during a survivorship time period where surveillance imaging is not typically warranted.

**Methods:**

Young adults aged 20–44 diagnosed with invasive malignancy in Ontario from 1992–1999 who lived at least 5 years from diagnosis were identified using the Ontario Cancer Registry and matched 5 to 1 to randomly selected cancer-free persons. We determined receipt of 5 modalities of diagnostic imaging and associated radiation dose received by survivors and controls from years 5–15 after diagnosis or matched referent date through administrative data. Matched pairs were censored six months prior to evidence of recurrence.

**Results:**

20,911 survivors and 104,524 controls had a median of 13.5 years observation. Survivors received all modalities of diagnostic imaging at significantly higher rates than controls. Survivors received CT at a 3.49-fold higher rate (95 % Confidence Interval [CI]:3.37, 3.62) than controls in years 5 to 15 after diagnosis. Survivors received a mean radiation dose of 26 miliSieverts solely from diagnostic imaging in the same time period, a 4.57-fold higher dose than matched controls (95 % CI: 4.39, 4.81).

**Conclusions:**

Long-term survivors of young adult cancer have a markedly higher rate of diagnostic imaging over time than matched controls, imaging associated with substantial radiation exposure, during a time period when surveillance is not routinely recommended.

**Electronic supplementary material:**

The online version of this article (doi:10.1186/s12885-015-1578-1) contains supplementary material, which is available to authorized users.

## Background

Epidemiologic evidence has established that exposure to ionizing radiation is a risk factor for leukemia and several solid cancers, with exposures at a younger age conferring a greater risk than later exposure [1–3]. Data suggest that acute exposure to 10–50 millisievert (mSv) or protracted exposure to 50–100 mSv of x- or γ-radiation infers an increased risk [4]. Studies have also demonstrated a positive association between diagnostic radiation and cancer risk [5–7]; repetitive computed tomography (CT) imaging and exposure during young adulthood may be particularly harmful [8–12]. The radiation dose associated with a CT study does not pose immediate risks; however, patients undergoing repeated CT studies accumulate radiation exposure over time. Some authors estimate that 29,000 future cancers could be related to the CT scans performed in 2007 in the United States alone [13].

Approximately 10,000 young adults (aged 20–44) are diagnosed with cancer annually in Canada [14]. Young patients are more radiosensitive than older adults and recent evidence has demonstrated that genetic factors may further heighten the association between diagnostic radiation and cancer risk in some groups [7, 15] (although not all young adults may have an increased genetic risk for developing cancer). In the example of breast cancer, young carriers of the BCRA 1 and 2 mutations may experience an increased risk of breast cancer at radiation dose levels considerably lower than those associated with an increased breast cancer risk in other cohorts exposed to radiation [15].

Patients diagnosed with a malignancy at a young age who survive have a substantial life expectancy and  cumulative exposure to diagnostic radiation will increase as they age. Patients may be exposed to low doses of radiation from various types of radiological studies used during initial diagnostic workup, and treatment monitoring. Additionally, surveillance guidelines for recurrence after initial treatment often rely on routine imaging (chest x-ray, CT) [16–19] for up to 5 years following treatment ( depending on malignancy and risk of recurrence) adding to lifetime radiation exposure. The routine use of diagnostic imaging for surveillance after 5 years is generally of little benefit since for most cancers late recurrence is uncommon [20–24], and  routine imaging may not be superior to clinical examination and evaluation of symptoms [25–28]. Little is known about patterns of diagnostic imaging among cancer survivors and to our knowledge, no study has evaluated this on a population basis among a young adult population at risk for accumulated radiation exposure from repetitive imaging over their lifetime .

We designed this study to investigate the uptake of diagnostic imaging and estimate cumulative diagnostic radiation exposure among a cohort of long-term survivors of young adult cancer compared to non-cancer controls in Ontario, Canada.

## Methods

The Research Ethics Board of St. Michael’s Hospital, Toronto, Ontario, Canada approved the study.

### Study design and setting

We designed a population-based retrospective cohort study using four data sources: the Ontario Cancer Registry (OCR), the Canadian Institute for Health Information Discharge Abstract Database (CIHI-DAD), the Ontario Health Insurance Plan (OHIP) database and the Registered Persons Database (RPDB).

OCR is a provincial cancer registry that has recorded all patients with incident cancers diagnosed in Ontario since 1964. Reporting to the OCR is provincially mandated and estimated to be 95 % complete [29]. CIHI-DAD contains information on all discharges from acute care hospitals and same day surgery units for residents of Ontario since April 1988. The OHIP database contains all claims for physicians and laboratory services provided to Ontario residents since 1991, essentially capturing the use of all physician services in Ontario. The RPDB is a roster of all individuals eligible for OHIP. All diagnostic codes in OCR are recorded according to the International Classification of Diseases.

### Selection of survivors

We used the OCR to identify all young adults, aged 20–44 at diagnosis of incident invasive malignancy (Additional file 1: Table S1) between January 1st, 1992 and December 31st, 1999. Patients were excluded if they had a previous malignancy, died within 5 years from diagnosis, were eligible for OHIP less than 7 years after diagnosis date, or had evidence of recurrent disease within 5 years of diagnosis. Recurrence is not recorded in the OCR; we modified a previously validated algorithm [30] that considered diagnosis of metastatic disease, receipt of palliative care or new chemotherapy found in administrative data to be evidence of recurrence (Additional file 1: Table S2). The recurrence date was defined as the earliest date of any palliative, chemotherapy or metastatic codes identified.

### Selection of controls

A cohort of matched controls was used to compare rates of imaging in survivors to the general population. Potential control subjects were selected from the general population using the RPDB, excluding those with a previous malignancy, matched to survivors by calendar year of birth, sex, and geographical region. Rates of diagnostic imaging in populations with cancer compared with the general population have showed differences based on these variables [31–33]. Potential controls were assigned a referent date corresponding to the date of the incident malignancy of their matched survivors; they were excluded if they died within 5 years of the referent date or if they became ineligible for OHIP in year 6 or 7 for reasons other than death. From the remaining potential controls, up to 5 were randomly selected without replacement for each survivor.

The cohort pairs were followed for a maximum of 15 years. Survivors who developed recurrent disease after 5 years of survivorship, along with their matched controls, were censored 6 months prior to the date of the first evidence of recurrence as the exact date of recurrence diagnosis was not obtainable. After 5-year survival, cohort pairs were additionally censored for death, end of OHIP eligibility or end of December 2010 for any pair member, whichever occurred first.

### Diagnostic imaging utilization

We identified OHIP professional billing codes [34] for CT (28 codes), plain radiography (171 codes), nuclear medicine (144 codes), MRI (11 codes) and diagnostic ultrasound (74 codes) (Additional file 1: Table S3). All CT (inpatient and outpatient) imaging is captured in OHIP. For other diagnostic imaging modalities, only outpatient imaging is captured. The date and number of diagnostic studies received by cohort members from the 5th year of survival through year 15 after diagnosis/referent date were identified in the OHIP database. Ultrasounds for fetal assessment were excluded to control for potential different rates of pregnancy in survivors and controls. If multiple imaging studies were billed on the same day, we included all procedures. Abdominal and pelvic CTs billed on the same day were considered a single abdo-pelvic CT.

We calculated the mean number of diagnostic studies received per person year for years 5 through 15 after the diagnosis/referent date for each imaging modality for survivors and controls. We also identified the physician specialty responsible for ordering CT scans in our population.

### Radiation dose

Effective dose is a commonly used metric providing a measure of harm from diagnostic radiation taking into account weighted averages of specific organ radiation dose according to the sensitivity of each organ to radiation [www.icrp.org/docs/Histpol.pfd]. We identified effective dose estimates for CT, plain radiography and nuclear medicine studies from current radiology literature and standard dosing references [35–37]. (Additional file 1: Table S3) Effective dose estimates used in this study are comparable with the range of published estimates for Canada and the United States and remained consistent over the observation period [38, 39].

### Statistical analysis

We calculated diagnostic imaging study rates as the number of diagnostic imaging studies per person-year of follow-up, overall, by type of imaging modality and by malignancy for survivors and controls. We used Poisson models for count data to compare rates of imaging studies in survivors versus controls, overall and by imaging modality, controlling for survivor status (survivor or control), malignancy type and socioeconomic status (SES), using an offset, the person-years of follow-up. We accounted for matching among survivors and controls by including a term for matched pairs. We did not find evidence of over-dispersion in the count data.

Mean and median cumulative effective dose (CED) received were calculated on an individual basis by tallying individual effective doses for all radiation-associated imaging studies received in years 5–15. CED was highly skewed, so it was log transformed for analysis. We compared the CED between survivors and controls using log-linear regression, adjusting for SES and malignancy type, and adjusting for differential follow-up times among pairs by weighting by person-years. Regression estimates were transformed back to the original scale and interpreted on a relative scale, presented by overall cancer and stratified by malignancy type with 95 % confidence intervals (CI).

We used SAS version 9.2 (SAS Institute, Cary, NC) for all statistical analyses. All statistical tests were 2-sided and significance was set at *P* <0.05.

## Results

**Fig. 1:**
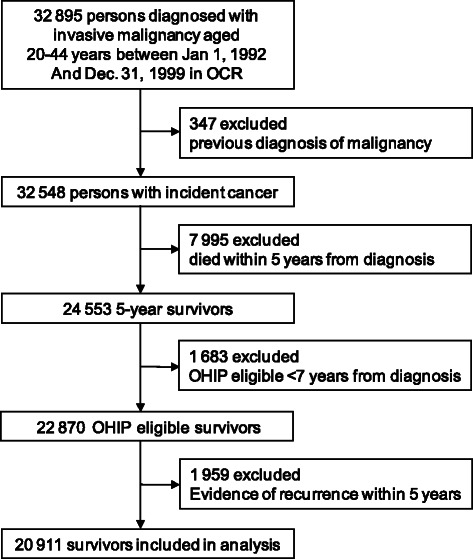
Cohort of survivors of young adult cancer with exclusions. OCR = Ontario Cancer Registry, OHIP = Ontario Health Insurance Plan

Between January 1st, 1992 and December 31st, 1999, 32,895 Ontarians age 20 to 44 were diagnosed with an invasive malignancy. Of these, 20,911 (63.6 %) were included in the analysis, with reasons for exclusion illustrated in Fig. 1. We identified 104,524 matched controls. The cohort distribution by sex and diagnoses are in Table 1. Survivors and controls had a median follow-up time of 13.5 (interquartile range [IQR] = 11.5, 15.6) years from date of diagnosis/reference. According to our administrative data algorithm, 1,199 (7.2 %) of YAS had evidence of recurrence after 5-year survival. Overall, 1,270 (6.1 %) survivors died in the observation period of the study (5–15 years after diagnosis/referent date); 836 (4.0 %) had evidence of recurrence and were censored at a median of 1.5 years before death. 429 survivors (2.1 %) died without evidence of recurrence, compared to 1,600 (1.5 %) deaths in the control group.

**Table 1:** Description of survivors of young adult malignancies and matched controls cohort at diagnosis/referent dates

	Survivors	Controls
	(n = 20,911)	(n = 104,524)
Age, mean (SD)	36 (6.3)	36 (6.3)
Median Follow-up Years^a^ (IQR)	13.5 (5.0,18.0)	13.5 (5.0,18.0)
Sex		
Male	6,801 (32.3)	33,997 (32.3)
Female	14,110 (67.7)	70,527 (67.7)
Malignancy Type		
Breast	4581 (21.9)	-
Gynecologic^b^	2782 (13.3)	-
Thyroid	2388 (11.4)	-
Melanoma	2088 (10.0)	-
Testicular	1390 (6.6)	-
Non-Hodgkin Lymphoma	1277 (6.1)	-
Hodgkin Lymphoma	1072 (5.1)	-
Urologic	895 (4.3)	-
Other	887 (4.2)	-
Colorectal	785 (3.8)	-
Head & Neck	768 (3.7)	-
Brain	632 (3.0)	-
Bone & Soft-tissue	463 (2.2)	-
Chest & Lung	345 (1.6)	-
Leukemia	284 (1.4)	-
Upper Gastrointestinal	274 (1.3)	-
Received Radiation	6,517 (32.1)	
Evidence of Recurrence	1,199 (7.2)	-
Died^c^	1,270 (6.1)	1,600 (1.5)
Income Quintile		
1 (lowest)	3,983 (19.0)	20,709 (19.8)
2	4,082 (19.5)	20,701 (19.8)
3	4,176 (20.0)	21,084 (20.2)
4	4,425 (21.2)	21,604 (20.7)
5 (highest)	4,245 (20.3)	20,426 (19.5)

Percentages reported in parenthesis, except for age and median follow -up time

^a^From date of diagnosis/reference.

^b^Includes ovarian, uterine and cervical malignancies.

^c^After 5-year survival.

Abbreviations: SD, standard deviation; IQR, interquartile range

### Patterns of diagnostic imaging

A total of 375,293 imaging studies were performed among survivors in the observation period; most were plain radiographs (48 %), diagnostic ultrasounds (31 %), or CT scans (12 %) (Table 2). The number of diagnostic studies received by survivors varied by malignancy type. For example, the mean number of CT scans was highest in survivors of non-Hodgkin lymphoma (NHL) (0.73 CT scans per person year) and lowest for survivors of thyroid cancer (0.15 CT scans per person year) (Table 2). Hematologists/oncologists were responsible for ordering the majority of CT scans (37 %) among survivors, while primary care practitioners were responsible for ordering the majority (38 %) among controls. A small proportion of CT scans were ordered by emergency family physicians among survivors (3 %) compared to 9 % ordered among controls.

**Table 2:** Mean number of diagnostic imaging studies received per person year, years 5–15 after diagnosis/referent dates, stratified by imaging modality and malignancy type

	CT		Plain Radiography		Nuclear Medicine		MRI		Ultrasound	
Malignancy Type	Survivors	Controls	Survivors	Controls	Survivors	Controls	Survivors	Controls	Survivors	Controls
All Malignancies	0.30	0.08	1.02	0.63	0.12	0.06	0.09	0.04	0.65	0.47
Thyroid	0.15	0.08	0.80	0.62	0.12	0.06	0.06	0.04	0.87	0.57
Melanoma	0.17	0.07	0.67	0.57	0.06	0.06	0.06	0.04	0.50	0.43
Gynecologic^a^	0.21	0.09	0.87	0.71	0.08	0.07	0.05	0.04	0.71	0.58
Bone & Soft-tissue	0.23	0.07	1.00	0.48	0.09	0.04	0.11	0.03	0.52	0.41
Head & Neck	0.25	0.09	0.76	0.53	0.09	0.07	0.13	0.03	0.42	0.37
Brain	0.26	0.08	0.58	0.50	0.06	0.05	0.65	0.03	0.39	0.84
Breast	0.32	0.10	1.70	0.84	0.22	0.08	0.10	0.04	0.85	0.59
Colorectal	0.33	0.08	0.87	0.62	0.09	0.07	0.05	0.04	0.64	0.42
Chest & Lung	0.33	0.10	1.13	0.60	0.09	0.06	0.04	0.03	0.43	0.41
Leukemia	0.34	0.07	0.91	0.49	0.09	0.06	0.06	0.04	0.60	0.73
Urologic	0.34	0.09	0.99	0.55	0.14	0.07	0.08	0.04	0.80	0.38
Testicular	0.40	0.07	0.68	0.34	0.06	0.04	0.03	0.03	0.29	0.20
HL	0.45	0.07	0.88	0.41	0.09	0.04	0.06	0.03	0.49	0.37
Upper GI	0.45	0.09	0.80	0.58	0.11	0.08	0.07	0.04	0.64	0.43
NHL	0.73	0.09	0.90	0.54	0.11	0.06	0.07	0.04	0.56	0.41

^a^Includes cervical, uterine and ovarian malignancies

Abbreviations: CT, computed tomography; MRI, magnetic resonance imaging; HL, Hodgkin lymphoma; GI, gastrointestinal; NHL, non-Hodgkin lymphoma

**Fig. 2:**
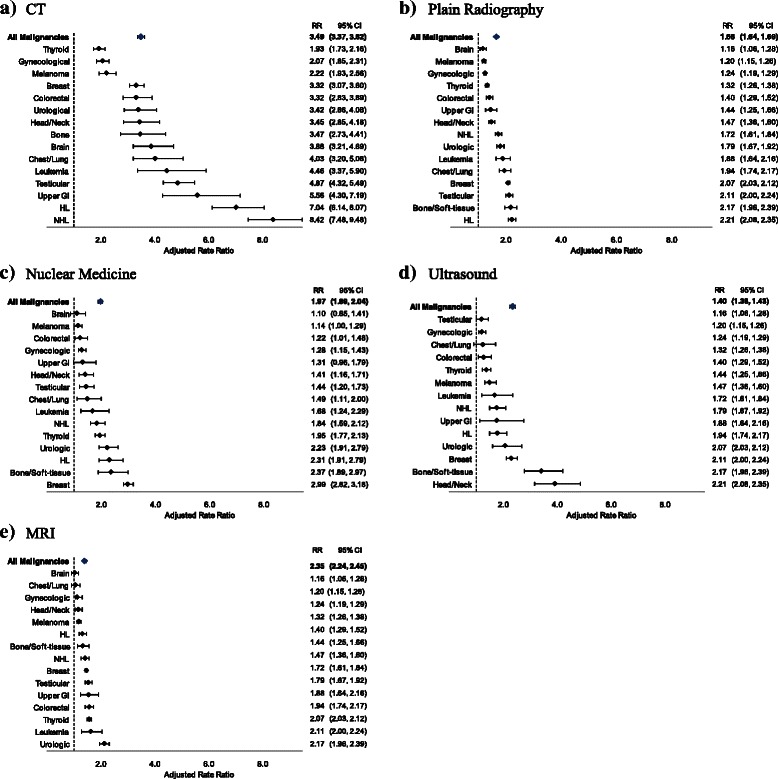
Adjusted rate ratios of diagnostic imaging associated with radiation (**a-c**) and not associated with radiation (**d-e**) received by young adult survivors of cancer compared to non-cancer controls in years 5–15 after diagnosis, by malignancy type. Note: RR of MRI for brain malignancy not shown in (e): RR = 22.04, 95 % CI: 19.21, 25.28. RR = rate ratio, CI = confidence interval, CT = computed tomography, MRI = magnetic resonance imaging, GI = gastrointestinal, HL = Hodgkin lymphoma, NHL = non-Hodgkin lymphoma

Survivors received all types of diagnostic studies at significantly higher rates than sex-, age- and geographically-matched controls. Overall, survivors received CT at an adjusted 3.49-fold higher rate than controls (rate ratio [RR] =3.49, 95 % CI: 3.37, 3.62, Fig. 2a). When stratified by malignancy type, all groups had a significantly higher rate of CT scanning than matched controls. Rate ratios ranged from 1.93 (thyroid, 95 % CI: 1.73, 2.16) to 8.42 (NHL, 95 % CI: 7.48, 9.48) (Fig. 2a). Rates of plain radiography (Fig. 2b) and nuclear medicine tests (Fig. 2c) were also higher in YAS as compared with controls.

Imaging studies not associated with radiation exposure were also more common in the survivor group. Survivors underwent ultrasound at a rate 1.4-fold higher (95 % CI: 1.38,1.43) (Fig. 2d) and MRI at a rate 2.35-fold higher (95 % CI: 2.24, 2.45) than matched controls (Fig. 2e). The rate of MRI was low in all malignancy groups except survivors of brain malignancies (RR = 22.04, 95 % CI: 19.21, 25.28; not shown in Fig 2e).

### Diagnostic radiation exposure

**Fig. 3:**
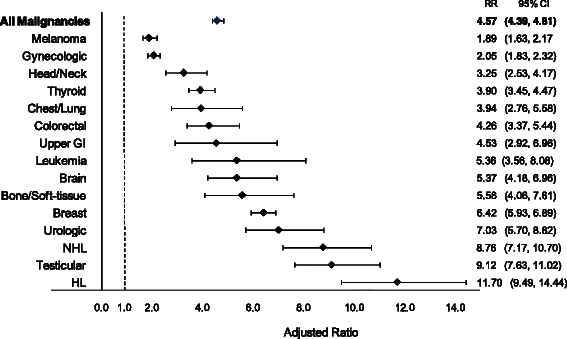
Adjusted cumulative effective dose (mSv) received by survivors of young adult cancer compared to controls in years 5–15 after diagnosis/referent dates, stratified by malignancy type. RR = rate ratio, CI = confidence interval, GI = gastrointestinal, HL = Hodgkin lymphoma, NHL = non-Hodgkin lymphoma.

Survivors received a mean CED of 26.3 mSv (median = 8.4 mSv) in years 5–15 after diagnosis, whereas controls received a mean dose of 10.7 mSv (median = 2.0 mSv) (Table 3). Overall, 16 % of survivors received a CED of 50 mSv or greater in this 10-year period (Table 3), with some malignancy groups receiving particularly high doses. Among survivors of non-Hodgkin lymphoma (NHL), almost a third exceeded the 50 mSv threshold and 15 % received ≥100 mSv in a 10-year period. Almost a quarter of survivors of gastrointestinal cancer and a fifth of survivors of breast, urologic, Hodgkin’s lymphoma and leukemia malignancies received ≥50 mSv. After adjusting for covariates, survivors received a 4.57-fold higher cumulative dose than matched controls (95 % CI: 4.39, 4.81) in years 5–15 after diagnosis (Fig. 3). When stratified by malignancy type, the CED of survivors ranged from 1.89-fold (melanoma, 95 % CI: 1.63, 2.17) to 11.70-fold higher (HL, 95 % CI: 9.49, 14.44) than matched controls (Fig. 3).

**Table 3:** Mean effective dose received by survivors and controls with proportion of survivors receiving 50 and 100 mSv in years 5 through 15 after diagnosis/referent dates, stratified by malignancy type

		Mean Effective Dose, mSv		Survivors Receiving Effective Dose			
		Dose, mSv		n (%)			
Malignancy Type	n	Survivors	Controls	50 mSv		100 mSv	
All Malignancies	20911	26.3	10.7	3333	(15.9)	1314	(6.3)
Breast	4581	34.4	13.0	909	(19.8)	386	(8.4)
Gynecologic^a^	2782	19.6	11.2	308	(11.1)	120	(4.3)
Thyroid	2388	18.3	10.0	258	(10.8)	60	(2.5)
Melanoma	2088	14.9	9.9	166	(8.0)	60	(2.9)
Testicular	1390	23.4	7.8	241	(17.3)	57	(4.1)
NHL	1277	46.0	10.2	364	(28.5)	193	(15.1)
HL	1072	31.8	7.2	211	(19.7)	98	(9.1)
Urologic	895	31.6	11.2	175	(19.6)	76	(8.5)
Colorectal	785	26.4	12.1	140	(17.8)	50	(6.4)
Head & Neck	768	22.5	11.4	101	(13.2)	40	(5.2)
Brain	632	16.3	9.4	47	(7.4)	8	(1.3)
Bone & Soft-tissue	463	20.8	7.9	64	(13.8)	24	(5.2)
Chest & Lung	345	28.2	11.3	66	(19.1)	29	(8.4)
Leukemia	284	26.8	8.9	55	(19.4)	28	(9.9)
Upper GI	274	36.7	12.0	68	(24.8)	25	(9.1)

^a^Includes cervical, uterine and ovarian malignancies.

Abbreviations: mSv, miliSieverts; NHL, non-Hodgkin lymphoma; HL, Hodgkin lymphoma; GI, gastrointestinal.

## Discussion

In this population-based cohort study, we showed that long-term survivors of young adult malignancies (greater than 5 years from diagnosis) received all types of diagnostic imaging, including CT associated with high effective doses of radiation, at significantly higher rates than controls in a time period where routine diagnostic imaging is not generally part of surveillance. Approximately one fifth of survivors received over 50 mSv of diagnostic radiation, with some groups, such as survivors of lymphoma, gastrointestinal, leukemia and urologic malignancies, receiving particularly high doses. The survivor cohort had no evidence of recurrence based on administrative data for 5 years after diagnosis and survivors were censored 6 months before any evidence of recurrence; therefore the excess imaging in survivors was unlikely to be related to the diagnosis of actual recurrence.

Few studies have investigated the uptake of diagnostic imaging among cancer survivors. These have focused generally on a specific type of radiologic investigation (e.g. mammography) [40], older patients [41] or earlier years of survival [42, 43]. Although recommendations for surveillance for recurrence after a primary cancer vary according to malignancy type, CT (when recommended) is used routinely when the risk of recurrence is highest, in general, during the first 5 years of survivorship [16, 44]. Routine surveillance is not recommended after 5-year survival for any malignancy based on current guidelines [16, 17, 45, 46]. While diagnostic imaging may be relied upon to detect recurrence after 5 years of recurrence-free survival, studies in both pediatric and adult patients with HL have suggested that relapses are symptomatic and that routine CT, in addition to being expensive, has poor specificity and provides minimal overall survival benefit [47, 48]. We found that the recurrence-free survivors of HL in our cohort received an average of 1 CT scan every 2 years in years 5–15 after diagnosis.

The estimates of diagnostic radiation exposure from this study are limited to years following 5-years from initial diagnosis, so many persons will have been exposed to considerably higher doses after considering imaging for initial diagnosis and evaluation and radiotherapy for primary treatment. Some persons may also be more susceptible to the effects of radiation, reinforcing the importance to minimize any exposure when possible. Survivors of gastrointestinal, urologic and leukemia malignancies in the current study were exposed to high doses of diagnostic radiation with approximately one fifth of survivors reaching a 50 mSv threshold within a 10 year time period. Notably, 15 % of NHL survivors were exposed to at least 100 mSv in the same time period. Survivors of testicular, upper gastrointestinal and lymphomas had at least a 4-fold higher rate of CT than controls. Even some survivors with a very low risk of recurrence, such as thyroid cancer, had more than a 2-fold higher rate of CT than controls. It is possible that this level of imaging may be appropriate; indications for diagnostic studies were not available in the administrative data used for this study. Hematologists/oncologists were responsible for ordering the majority of CT scans among survivors in this study. There were fewer scans ordered by emergency family physicians among survivors (3 %) than controls (9 %), suggesting that high CT rates among survivors are unlikely to be related to emergency care. However, identifying high rates of imaging highlights the need for future research directed at identifying potential elective diagnostic studies and ability to select alterative imaging modalities or non-imaging strategies to reduce radiation exposure whenever possible.

Although controversial, a number of epidemiologic studies have found significant associations between diagnostic radiation and the incidence of cancer [12, 15, 49, 50], highlighting the potential for harm associated with repetitive exposure to diagnostic imaging and CT in particular. Cancer patients receiving repetitive diagnostic and treatment monitoring imaging studies are at risk of high cumulative exposure in a short period of time. For example, 16 % of survivors in the current study received 50 mSv or more in a 10-year period. Survivors who received radiation therapy for primary treatment (30 %) are also at an additional risk of secondary carcinogenesis due to radiation exposure [51, 52]. The potential for harm associated with radiation exposure is cumulative; as survivors age and continue to be imaged, small effective doses will accumulate to even higher life-time exposures. Many survivors have had substantial effective doses from recommended surveillance CT scans up to 5 years post-treatment, emphasizing the importance of minimizing imaging after 5-year recurrence-free survival, when possible.

### Limitations

We used administrative data to identify receipt of imaging and therefore, could not determine the indication for imaging studies. Many studies may have been ordered for investigation of symptoms, but the symptoms were not due to disease recurrence. The OCR does not collect data on cancer recurrence, therefore we relied on an algorithm based on administrative codes. While a small number of survivors with recurrence may not have been detected by our algorithm, the rate of death without recurrence in survivors (2.1 %) was similar to the overall death rate of controls (1.5 %), indicating our algorithm successfully identified recurrent disease. Finally, because dosing metrics for imaging investigations were not available, we were not able to calculate absorbed doses and instead used effective dose. However, the use of a different metric would not influence the magnitude of difference found in exposure between survivors and controls.

The population-based and comprehensive data used for our study provided a unique opportunity to evaluate patterns of diagnostic imaging in all survivors in the province. Due to the lack of population-based health services data for similar groups of patients in many jurisdictions, our study would be difficult to replicate elsewhere, increasing the importance and relevance of our findings.

### Implications for future research

Determining the optimal use of imaging among survivors of young adult cancer is challenging; while these individuals are at risk of recurrence and second malignancies, these risks vary according to malignancy type and must be balanced against the risk of cumulative radiation exposure. Although a higher rate of imaging in some survivors as compared to the general population may be appropriate, we found an increased rate for survivors extending up to 15 years after diagnosis for all malignancies and for all imaging modalities, indicating likely over-use of imaging in these patients. Strategies to reduce radiation exposure in survivors, including (when possible) the substitution of diagnostic procedures not associated with radiation, such as ultrasound, and the cessation of surveillance after 5 years should be considered. Educational interventions among physicians and patients that encourage good stewardship in the use of imaging, particularly the use of imaging associated with significant radiation exposure, should be explored.

## Conclusions

Our study showed that survivors of young adult malignancies receive CT imaging studies and associated radiation at significantly higher rates than the general population during a time period where surveillance imaging is not typically recommended.

## Additional file

Additional file 1: Table S1. International classification of disease (ICD)-9 codes and descriptions of malignancy types. **Table S2.** Administrative data codes used for identifying evidence of recurrent disease in young adult cancer survivors. **Table S3.** Ontario Health Insurance Plan (OHIP) professional fee codes for plain radiography, nuclear medicine, computed tomography, ultrasound and magnetic resonance imaging studies and effectives dose estimates for imaging studies associated with diagnostic radiation used in this study. (PDF 92 kb)

## Abbreviations

CED: cumulative effective dose
CIHI-DAD: Canadian Institute for Health Information Discharge Abstract Database
CT: computed tomography
HL: Hodgkin lymphoma
GI: Gastrointestinal
MRI: Magnetic resonance imaging
mSv: Millisievert
NHL: Non-Hodgkinlymphoma
OCR: Ontario Cancer Registry
OHIP: Ontario Health Insurance Plan
RPDB: Registered Persons Database
RR: Rate ratio
SES: Socioeconomic status
