# Supplementary material for: Patterns of diagnostic imaging and associated radiation exposure among long-term survivors of young adult cancer: a population-based cohort study
Source: BMC Cancer. 2015 Sep 3;15:612. doi: 10.1186/s12885-015-1578-1 (PMC4559270; doi:10.1186/s12885-015-1578-1)
Supplement: Additional file 1: Table S1. — International classification of disease (ICD)-9 codes and descriptions of malignancy types. Table S2. Administrative data codes used for identifying evidence of recurrent disease in young adult cancer survivors. Table S3. Ontario Health Insurance Plan (OHIP) professional fee codes for plain radiography, nuclear medicine, computed tomography, ultrasound and magnetic resonance imaging studies and effectives dose estimates for imaging studies associated with diagnostic radiation used in this study. (PDF 92 kb) [file 12885_2015_1578_MOESM1_ESM.pdf]

Additional file 1

Table S1. International classification of disease (ICD)-9 codes and descriptions of malignancy types.

Malignancy Type	ICD-9	Description
Bone & Soft-tissue	170.0 - 170.9	Malignant neoplasm of bone and articular cartilage
	171.0 - 171.9	Malignant neoplasm of connective and other soft tissue
	158.0	Retroperitoneium
	203.0	Multiple Myeloma
Brain	191.0 - 191.9	Malignant neoplasm of brain
	192.0 - 192.9	Malignant neoplasm of other and unspecified parts of Nervous System
Breast	174.0 - 174.9	Malignant neoplasm of female breast
Chest & Lung	162.0 - 162.9	Malignant neoplasm of trachea, bronchus and lung
	163.0 - 163.9	Malignant neoplasm of pleura
	164.0 - 164. 9	Malignant neoplasm of thymus, heart and mediastinum
Colorectal	153.0 - 153.9	Malignant neoplasm of colon (excludes 153.5, appendix)
	154.0 - 154.1	Malignant neoplasm rectosigmoid junction and rectum
Gynecological	180.0 - 180.9	Malignant neoplasm of cervix uteri
	179, 182.0 - 182.2	Malignant neoplasm of uterus
	183.0 - 183.9	Malignant neoplasm of ovary and other uterine adnexa
Head & Neck	140.0 - 140.9	Malignant neoplasm of lip
	149.0 - 149.9	Malignant neoplasm of other and ill-defined sites within the lip, oral cavity and pharynx
	160.0 - 160.9	Malignant neoplasm of nasal cavities, middle ear, and accessory sinuses
	161.0 - 161.9	Maligant neoplasm of larynx
Hodgkin Lymphoma	201.0 - 201.9	Hodgkin's disease
Leukemia	204.0 - 204.9	Lymphoid leukaemia
	205.0 - 205.9	Myeloid leukaemia
	206.0 - 206.9	Monocytic leukaemia
	207.0 - 207.9	Other specified leukaemia
	208.0 - 208. 9	Leukaemia of unspecified cell type
Melanoma	172.0 - 172.9	Malignant melanoma of skin
Non-Hodgkin's Lymphoma	200.0 - 200.8	Lymphosarcoma and reticulosarcoma
	202.0 - 202.9	Other malignant neoplasm of lymphoid and histiocytic tissue
Other	154.2	Anal canal
	154.3	Anus, unspecified
	154.8	Anus, other
Testicular	186	Malignant neoplasm of testis
Thyroid	193	Malignant neoplasm of thyroid gland
Upper Gastrointestinal	150.0 - 150.9	Malignant neoplasm of oesophagus
	151.0 - 151.9	Malignant neoplasm of stomach
	152.0 - 152.9	Malignant neoplasm of small intestine, including duodenum
	153.5	Appendix
	155.0 - 155.2	Malignant neoplasm of liver and intrahepatic bile ducts
	156.0 - 156.9	Malignant neoplasm of gallbladder and extrahepatic bile ducts
	157.0 - 157.9	Malignant neoplasm of pancreas
Urologic	188.0 - 188.9	Malignant neoplasm of bladder
	189.0 - 189.9	Malignant neoplasm of kidney and other and unspecified urinary organs

Table S2. Administrative data codes used for identifying evidence of recurrent disease in young adult cancer survivors.

**Criteria**		**Database**	**Codes**
Palliative Care	Family Practise	OHIP	A945, C882, C945, K023, W872, W882
	Community Medicine	OHIP	W972, W982
	Anaiesthesia	OHIP	C982
Chemotherapy		OHIP	G281, G339, G345, G359, G381, G390
Metastatic Disease	ICD-9	CIHI	196.0, 196.1, 196.2, 196.5, 196.8, 196.9, 197.0, 197.1, 197.2, 197.3, 197.4, 197.5, 197.6, 197.7, 197.8, 198.0, 198.1, 198.2, 198.3, 198.4, 198.5, 198.6, 198.7, 198.8, 199.0, 199.1
	ICD-10	CIHI	C770, C771, C772, C774, C775, C778, C779, C780, C781, C782, C783, C784, C785, C786, C787, C788, C790, C791, C792, C793, C794, C795, C796, C797, C798

Abbreviations: OHIP, Ontario Health Insurance Plan; ICD, International Classification of Disease; CIHI, Canadian Institute for Health Information.

Table S3. Ontario Health Insurance Plan (OHIP) professional fee codes for plain radiography, nuclear medicine, computed tomography, ultrasound and magnetic resonance imaging studies and effectives dose estimates for imaging studies associated with diagnostic radiation used in this study.

Body Part	Feecode(s)	ED, mSv
Plain Radiography		
**Head and Neck**		
Skull, facial bones, nose, mandible, sinuses, mastoids, neck, other	X001, X009, X003, X004, X005, X006, X012, X007, X008, X010, X011, X016, X017, X018, X019, X020	0.1
**Spine and Pelvis**		
Cervial spine	X025, X202, X203	0.2
Pelvis and/or hips, 1 view	X036	0.7
Pelvis and/or hips, 2 views	X037	1.4
Pelvis and/or hips, 3 views	X038	2.1
Sacro-iliac joints	X035, X208	0.83
Thoracic spine	X027, X204	1
Lumbar or lumbosacral spine, 2 or 3 views	X028	1.5
Lumbar or lumbosacral spine, 4 or 5 views	X205	2
Lumbar or lumbosacral spine, 6 or more views	X206	2
Entire spine (scoliosis series)	X032, X033, X031	2.5
Sacrum and/or coccyx, 2 views	X034	2
Sacrum and/or coccyx, 3 or more views	X207	3
**Upper Extremities**		
Clavicle, acromioclavicular joints, sternoclavicular joing, scapula, humerus, elbow, forearm, wrist, hand, finger, thumb	X045, X209, X046, X210, X047, X211, X049, X213, X050, X214, X051, X215, X216, X052, X217, X218, X053, X054, X219, X055, X220, X056, X221	0.001
Shoulder	X048, X212	0.01
**Lower Extremities**		
Femur (including one joint), tibia, fibula, ankle, calcaneus, foot, toe, leg length studies (orthoroentgenogram)	X063, X223, X066, X226, X067, X227, X068, X228, X069, X229, X072, X230, X064	0.001
Knee including patella	X065, X224, X225	0.005
Hip (unilateral)	X060	0.07
**Skeletal Surveys**		
Survey for bone age (single film), other surveys (e.g. rhematoid, metabolic, metastatic)	X057, X080, X081	3.5
Survey for bone age (2 or more films)	X058	7
**Chest and Abdomen**		
Chest, single view	X090	0.02
2 views of chest, sternum or thoracic inlet	X091, X040, X096	0.1
Chest (3 or more views), ribs	X092, X039	0.2
Abdomen (1 view)	X100	0.7
Abdomen (2 or more views)	X101	1.4

**Gastrointestional Tract**		
T-tube, operative cholangiogram, operative pancreatogram	X116, X117, X123	4.0
Oesophagus, stomach, duodenum (with double contrast)	X103, X109	6.0
Gallbladder (one exam)	X114	1.0
Gallbladder (with preliminary plain film)	X120	2.0
Oesophagus, stomach, duodenum, small bowel	X108, X104, X110, X111	3.0
Colon, barium enema	X112, X113	7.0
Palatopharyngeal analysis, pharynx, oesophagus	X105, X106, X107	
**Genitoury Tract**		
Intravenous pyelogram, cystourethrogram, nephrostogram, percutaneous pyleogram, percutaneous nephrostogram	X130, X135, X131, X191, X138, X139	6.0
Retrograde urethrogram, vasogram, cavernosography	X134, X136, X141	2.0
Retrograde pyelogram, cystogram	X129, X137	3.0
**Obstetrics & Gynaecology**		
Hysterosalpingogram	X147	4.0
**Fluoroscopy**		
Chest, skeleton, abdomen	X189, X195, X196, X197	3.0
**Bone Mineral Density (BMD) Measurement**		
Baseline, second test or subsequent test	X145, X146, X152, X153, X142, X148, X149, X155	0.0013
**Special Examinations**		
Selective or non-selective (using film charger) abdominal, thoracic, cervical or cranial angiogram by catherization	X180, X181, X182	20.0
Abdominal, thoracic, cervical or cranial angiogram by catherization (single fim)	X179	12.0
Selective (5 or more vessels) abdominal, thoracic, cervical or cranial angiogram by catherization	X140	40.0
Cholangiogram	X122	6.0
Vertebral angiogram - direct puncture or brachial injection	X132, X133	
Cerebral stereotaxis, spleoportogram, arthrogram	X162, X121, X198, X199, X156, X200	
Carotid angiogram by direct puncture	X160, X161	
Peripheral angiogram	X174, X175	
Bronchogram	X158, X159	
**Miscellaneous Examinations**		
Mammogram, mammary ductography, breast biopsy specimen	X172, X178, X184, X185, X192, X194, X201	0.4
Myelogram -spine and/or posterior fossa	X173	6.0
Other	X164, X167, X169, X170, X171, X150, X193, X190, X154, X165, X176, X177, X183, X151, X163	

Nuclear Medicine		
**Cardiovascular System**		
Venography, cardioangiography, myocardial perfusion scinitigraphy, myocardial wall motion, other	J802, J602, J804, J604, J867, J667, J806, J606, J807, J607, J808, J608, J810, J610, J811, J611, J812, J612, J813, J613, J814, J614, J815, J615	12.55
**Endocrine System**		
Adrenal, thyroid scintigraphy	J816, J616, J868, J668, J869, J669, J818, J618	4.8
Thyroid scintigraphy	J871, J671, J817, J617, J870, J670	1.9
Parathyroid scintigraphy	J820, J620	6.7
Other (with I-131)	J872, J672	10.0
**Gastrointestinal System**		
Schilling Test	J821, J621, J823, J623	0.162
Malabsorption Test	J824, J624, J873, J673	0.003
Gastrointestinal (with Cr-52)	J874, J674	0.943
Liver/spleen scintigraphy	J832, J632	2.1
Biliary scintigraphy	J831, J631	3.1
Abdominal scintigraphy for intestinal bleed	J830, J630, J878, J678, J879, J679	7.8
Calcium Absorption	J826, J626, J875, J675, J827, J627	
Other gastrointestinal (protein loss, transit or reflux study)	J825, J635, J829, J629	
Gastro-oesophageal	J876, J676, J877, J677	
Salivary gland scintigraphy	J833, J633	
**Genitourinary System**		
Dynamic renal imaging or static renal scintigraphy	J834, J634, J836, J636	5.18
Computer assessed renal function	J835, J635, J880, J680	
Testicular and scrotal scintigraphy	J840, J640	
Other	J837, J637, J838, J638, J839, J639	
**Haematopoietic**		
Bone marrow scintigraphy	J881, J681, J882, J682	2.78
In-111 leukocyte scintigraphy	J883, J683, J884, J684	6.7
Plasma volume	J841, J641	
Red cell volume	J843, J643	
Red cell, white cell or platelet survival	J848, J648, J849, J649	
Other	J847, J647	
**Musculoskeletal System**		
Bone scintigraphy	J850, J650, J851, J651	6.3
Gallium Scintigraphy	J852, J652, J853, J653	15.0
**Nervous System**		
Cerebral fluid circulation, brain scintigraphy	J857, J657, J885, J685, J886, J686, J858, J658	6.88

**Respiratory System**		
Ventilation lung scintigraphy	J887, J687	0.5
Perfusion lung scintigraphy	J859, J659	2.0
Perfusion and ventilation scientigraphy	J860, J660	2.5
**Miscellaneous**		
Scintomammography	J863, J663	6.66
Radionuclide lymphangiogram, ocular tumour localization, tear duct scintigraphy	J861, J661, J862, J662, J864, J664, J865, J665	
Computed Tomography		
CT guidance of biopsy	X168	30.0
Abdomen with and without IV contrast,<br>pelvis with and without IV contrast	X126, X233	17.6
Cardio-thoracic CT (also known as CT angiography)	X235	15.0
Thorax with and without IV contrast	X125	14.0
Spine with and without IV contrast	X128	11.8
Abdomen with IV contrast,<br>abdomen without IV contrast,<br>pelvis with IV contrast,<br>pelvis without IV contrast	X409, X410, X231, X232	8.8
Thorax without IV contrast,<br>thorax with IV contrast	X406, X407	7.0
Neck with IV contrast	X404	6.0
Spine without IV contrast,<br>spine with IV contrast	X415, X416	5.9
Head with IV contrast,<br>complex head with and without IV contrast	X401, X408	4.0
Neck with and without IV contrast,<br>neck without IV contrast	X124, X403	3.0
Head with and without IV contrast,<br>head without contrast,<br>complex head with IV contrast,<br>complex head without IV contrast	X188, X400, X402, X405	2.0
Extremities	X127, X412, X413	
CT perfusion	E874	
CT colonography	X234	

Diagnostic Ultrasound	
Head and Neck	J122, J422, J102, J402, J103, J403, J107, J407, J108, J408, J105, J405
Pelvis	J162, J462, J138, J438, J165, J476, J163, J463
Extra-cranial vessel assessment	J190, J490, J191, J491, J192, J492, J201, J501
Peripheral vessel assessment	J193, J493, J194, J494, J195, J495, J202, J502
Thorax, Abdomen and Retroperitoneum	J125, J425, J135, J435, J128, J428
Intracavitary	J161, J461, J164, J464
Echography	J180, J480
Extremities	J182, J482
Breast	J127, J427
Scotal	J183, J483
Transcranial	J189, J489
Venous Assessment	J198, J498
Doppler evaluation of organ transplantation	J205, J505
Duplex evaluation or portal hypertension	J206, J506
Duplex assessment of patency obstruction	J207, J507
Ankle Pressure Measurements	J200, J500
Penile pressure recordings	J197, J497
Penile Doppler evaluation	J199, J499
Transcutaneous tissue	J203, J503, J204, J504
Magnetic Resonance Imaging	
Head	X421
Neck	X431
Thorax	X441
Breast	X446
Abdomen	X451
Pelvis	X461
Extremities	X471, X488
Spine	X490, X493, X496

Abbreviations: ED, efective dose; 1-131; iodine isotope 131; Cr-52, chromium isotope 52; In-111, indium isotope 111; CT, computed tomography.
